# *Lactobacillus paracasei* PS23 Delays Progression of Age-Related Cognitive Decline in Senescence Accelerated Mouse Prone 8 (SAMP8) Mice

**DOI:** 10.3390/nu10070894

**Published:** 2018-07-12

**Authors:** Shih-Yi Huang, Li-Han Chen, Ming-Fu Wang, Chih-Chieh Hsu, Ching-Hung Chan, Jia-Xian Li, Hui-Yu Huang

**Affiliations:** 1 Graduate Institute of Metabolism and Obesity Sciences, Taipei Medical University, Taipei 11031, Taiwan; sihuang@tmu.edu.tw; 2 Department of Food Science, Nutrition, and Nutraceutical Biotechnology, Shih Chien University, Taipei 10462, Taiwan; lihan.h.chen@gmail.com (L.-H.C.); llfonly520@gmail.com (C.-H.C.); bless.me.you@gmail.com (J.-X.L.); 3 Department of Food and Nutrition, Providence University, Taichung 43301, Taiwan; mfwang@pu.edu.tw; 4 Biomedical Co., Ltd., Taipei 10448, Taiwan; cchsu@yangsen.com.tw

**Keywords:** aging, senescence-accelerated mouse prone 8 (SAMP8), *Lactobacillus paracasei* PS23, cognitive impairment

## Abstract

Probiotic supplements are potential therapeutic agents for age-related disorders due to their antioxidant and anti-inflammatory properties. However, the effect of probiotics on age-related brain dysfunction remains unclear. To investigate the effects of *Lactobacillus paracasei* PS23 (LPPS23) on the progression of age-related cognitive decline, male and female senescence-accelerated mouse prone 8 (SAMP8) mice were divided into two groups (*n* = 6 each): the control and PS23 groups. From the age of 16 weeks, these groups were given saline and LPPS23, respectively, because SAMP8 mice start aging rapidly after four months of age. After 12 weeks of treatment, we evaluated the effect of LPPS23 by analyzing their appearance, behavior, neural monoamines, anti-oxidative enzymes, and inflammatory cytokines. The PS23 group showed lower scores of senescence and less serious anxiety-like behaviors and memory impairment compared to the control group. The control mice also showed lower levels of neural monoamines in the striatum, hippocampus, and serum. Moreover, LPPS23 induced the anti-oxidative enzymes superoxide dismutase (SOD) and glutathione peroxidase (GPx). Higher levels of tumor necrosis factor (TNF)-α and monocyte chemotactic protein-1 (MCP1) and lower levels of interleukin (IL)-10 indicated that LPPS23 modulated the inflammation. Our results suggest that LPPS23 supplements could delay age-related cognitive decline, possibly by preventing oxidation and inflammation and modulating gut–brain axis communication.

## 1. Introduction

The average global lifespan is increasing. As the population is aging, the incidence of brain dysfunction, including neurodegenerative and neuropsychiatric disorders, is expected to increase rapidly [1]. Anxiety and memory deficit are two common symptoms of brain dysfunction caused by aging [2,3]. Although some studies have demonstrated methods to attenuate the progression of these symptoms [4,5,6], anxiety and memory deficit remain serious issues in the aged population. Therefore, the exploration of alternative treatments for age-related anxiety and memory impairment is necessary.

Inflammation and free radical levels are both correlated with the speed of aging [7,8] and therefore probiotic supplements may be a feasible treatment option owing to their diverse effects, namely anti-inflammatory activities [9], antioxidant activities [10], and metabolic regulation [11]. Recent studies have also suggested that probiotics use the gut–brain axis to increase the levels of neural monoamines, such as dopamine (DA), serotonin (5-HT), and brain-derived neurotropic factor (BDNF), which are critical for neuronal plasticity and survival [12]. Preventing decreases in the levels of DA, 5-HT, and BDNF is believed to reduce the prevalence of anxiety and memory deficits [13]. Since the levels decrease during aging [14,15], maintaining them should prevent age-associated cognitive declines.

Probiotics are widely believed to affect the central nervous system and behavior [16]. For example, *Lactobacillus helveticus*, *Bifidobacterium longum*, and *B. breve* reduced anxiety-like behaviors and enhanced memory in murine models [17,18,19]. These improved behaviors were accompanied by restoration of the neural monoamine levels in key regions of the brain, such as the hippocampus and striatum [20]. However, these experiments were only conducted using young and middle-aged adult animals. Thus, the effects of probiotics on age-related alterations in emotion and memory in aged animals remain unclear.

Aging-related studies are expensive and time consuming because the breeding period of experimental animals is relatively long. Therefore, prematurely aged animal models are often used in age-related research. Senescence-accelerated mouse prone 8 (SAMP8) is a line developed from the senescence-resistant mice (SAMR1) created by Takeda et al. (1981). It is characterized by an early onset of age-related alterations, such as hair loss, dull hair, and short lifespan [21]. SAMP8 mice are comparable to normal SAMR1 mice before four months of age, but SAMP8 mice exhibit significant emotional and memory impairments after six months of age; thus, the onset of impairments occurs considerably earlier in SAMP8 mice than in normal SAMR1 mice [22,23,24,25]. Rhea and Banks (2017) confirmed the appropriateness of SAMP8 mice as an animal model for studies on age-related emotion and memory dysfunction [26]. They also reported that SAMP8 mice start aging rapidly after four months of age. On the basis of their findings, we initiated the *L. paracasei* PS23 (LPPS23) supplementation at an early stage of aging (16-week-old) for 12 weeks in SAMP8 mice to further understand the effects of LPPS23 on the progression of aging.

LPPS23 was isolated from healthy human feces and deposited at the Leibniz Institut DSMZ-Deutsche Sammlung von Mikroorganismen und Zellkulturen GmbH with the accession number DSM 32322. Although LPPS23 has not been investigated, previous studies have suggested that *L. paracasei*, either alone or mixed with other probiotics, significantly affects the brain and nervous system [27,28,29]. Moreover, *L. paracasei* NTU 101 has been reported to prevent oxidative stress and modulate inflammatory responses [30,31]. Thus, we hypothesized that LPPS23 exhibits similar functions in SAMP8 mice.

In the present study, we aimed to investigate the preventive effect of LPPS23 on age-related cognitive decline (anxiety-like behaviors and memory impairment) in SAMP8 mice. Therefore, LPPS23 supplementation was started at 16-week-old before rapid aging started in SAMP8 mice [26]. After 12 weeks, the effect and mechanism of LPPS23 was evaluated by comparing the degree of change in senescence, behaviors, the levels of neuro-monoamines and inflammation, and free radical scavenging activity between the aged (control) and LPPS23 supplemented (PS23) SAMP8 mice.

## 2. Materials and Methods

### 2.1. L. paracasei PS23 and Experimental Animals

The culture of LPPS23 was provided by Professor Ying-Chieh Tsai (National Yang-Ming University, Taipei, Taiwan). The LPPS23 culture was subcultured, and the concentration was adjusted to 1 × 10^9^ CFU/200 μL for subsequent use. SAMP-8 mice were provided by Dr. Ming-Fu Wang (Providence University, Taichung, Taiwan) and housed under standard laboratory conditions (12/12-h light/dark cycle, 22–24 °C, 40–60% humidity). The 16-week-old SAMP8 mice were fed a commercially available diet (local supplier) and provided sterile water ad libitum. Male and female mice were divided into two groups each (*n* = 6), namely control (fed for 12 weeks and treated with saline until the age of 28 weeks) and PS23 (fed for 12 weeks and treated with LPPS23 [1 × 109 CFU/mouse/day] until the age of 28 weeks) groups. The dose of LPPS23 was chosen based on previous studies indicating that supplementing probiotics at 1 × 10^9^ CFU/mouse/day improved either anxiety or memory in mice [12,19,32,33]. To ensure that the mice received live LPPS23, bacteria were delivered by gavage performed by well-trained investigators. The control and PS23 groups were treated with either saline or LPPS23 by gavage at 9:00 a.m. daily for 12 weeks. The mice were sacrificed at end of 28 weeks after the evaluation of senescence and the open-field (OF) and Morris water maze (MWM) tests. Brain and serum samples of the mice were collected. All animal experiments were performed in accordance with protocols approved by the Institutional Animal Care and Use Committee (IACUC) of Shih Chien University (IACUC-10407).

### 2.2. Evaluation of Senescence

The degree of senescence was evaluated using a grading score system, as described previously [21]. Briefly, there were 11 categories of senescence related to reactivity, passivity, skin and hair, eyes, and spine. Each category had five grades ranging from 0 to 4. A higher grade indicates more serious senescence. Scores in each category were given by four investigators and added to obtain the grading score for each mouse. An average grading score was calculated for each group. The criteria for the grading score of senescence in mice are shown in Supplementary Table S1.

### 2.3. Open Field Test

Each mouse was placed in an OF activity chamber with plexiglas walls (25.4 × 25.4 × 38 cm) for 10 min (Tru Scan Activity System; Coulbourn Instruments, Whitehall, PA, USA). The total distance they walked was measured using the Ethovision video tracking software (Noldus, Wageningen, The Netherlands). To minimize odor interference, the chamber was cleaned with 70% ethanol after each run.

### 2.4. Morris Water Maze Test

The MWM test was performed in a circular pool (960 mm in diameter and 50 cm in height) containing water (25 ± 2 °C) filled to a depth of 40 cm. The mice were trained to find a platform below the surface of the water four times per day for 6 days. The mice were guided to climb on the platform if they could not escape to the platform within 60 s. On the seventh day, the platform was removed, and the mice were given 60 s to search the maze. The swimming path was analyzed using the Ethovision video tracking software (Noldus). The swimming time in the former platform quadrant and the total swimming time in all four quadrants were recorded for 60 s.

### 2.5. Quantification of Neuronal Amines and Their Metabolites

The striatum and hippocampus samples of the mice were collected, immediately stored in ice-cold 0.6% perchloric acid, and homogenized through sonication. The samples were centrifuged at 12,000 rpm for 20 min at 4 °C; the supernatants were filtered using a 0.22-µm membrane (4-mm syringe filter; Millipore, MA, USA) and stored at −80 °C until use. The levels of DA, 5-HT, and their metabolites were determined using a high-performance liquid chromatography–electrochemical detection system that consisted of a micropump (CMA-100; CMA, Stockholm, Sweden), an online injector (CMA-160), a Microtech LC-pump (Microtech Scientific, Sunnyvale, CA, USA), a BAS-4C electrochemical detector (Bioanalytical Systems, Inc., West Lafeyette, IN, USA), and a reverse-phase column (Kinetex C18, 2.6 µm, 100 × 2.1-mm internal diameter; Phenomenex, CA, USA), as described previously [34]. The potential of the glassy carbon working electrode was set at +650 mV with respect to the Ag/AgCl reference electrode at room temperature. The mobile phase contained 0.1 M NaH_2_PO_4_, 8% methanol, 0.74 mM 1-octanesulfonic acid (sodium salt), 0.03 mM ethylenediamine tetraacetic acid, and 2 mM KCl, and the pH was adjusted to 3.74 with H_3_PO_4_. The levels of DA, dihydroxyphenylacetic acid (DC), 5-HT, and 5-hydroxyindoleacetic acid (5-HIAA) were interpolated using a standard curve.

### 2.6. Enzyme-Linked Immunosorbent Assay

The levels of pro-inflammatory factors and brain-derived neurotrophic factor (BDNF) in the serum samples were determined using sandwich enzyme-linked immunosorbent assay (ELISA) kits for tumor necrosis factor (TNF)-α (Biolegend, San Diego, CA, USA), interleukin (IL)-10 (Peprotech, Rocky Hill, NJ, USA), and monocyte chemotactic protein-1 (MCP-1) (Biolegend) and the mouse BDNF ELISA kit (ScienCell, Carlsbad, CA, USA), respectively. All the assays were performed according to the manufacturer’s instructions. The serum samples were used without dilution. Absorbance values were measured using an ELISA reader (BioTek, Winooski, VT, USA).

### 2.7. Antioxidative Enzyme Activities

The activities of superoxide dismutase (SOD) and glutathione peroxidase (GPx) were assayed in the serum samples by using SOD and GPx assay kits (Cayman Chemicals Inc., Ann Arbour, MI, USA), respectively, following the manufacturer’s instructions. The activities of SOD and GPx were calculated using an equation obtained from the linear regression of the standard curve.

### 2.8. Statistical Analyses

Data and results are presented as the mean ± standard error of the mean. A one-tailed Student’s t-test was used to analyze the difference between the groups. A value of *p* < 0.05 was considered statistically significant.

## 3. Results

### 3.1. LPPS23 in the Aged SAMP8 Mice

**Figure 1:**
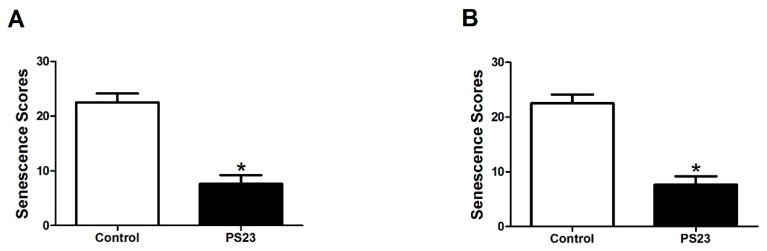
Grading scores of appearance senescence in the control and *L. paracasei* PS23 treated (PS23) groups in the (**A**) male and (**B**) female mice. Data were analyzed using the *t*-test. * *p* < 0.05.

To examine the effects of LPPS23 on aging and brain functions in both sexes of the control SAMP8 mice, the male and female mice were also analyzed individually. The degrees of senescence were evaluated using the grading score system described in the study conducted by Takeda et al. The score is positively correlated with the degree of senescence. The grading scores of the male and female SAMP8 mice in the control group were higher than those of the male and female mice in the PS23 group. The grading scores were 38% and 46% lower in the male and female mice, respectively, in the PS23 group than in the control group, as shown in Figure 1.

### 3.2. Behavioral Changes in the LPPS23-Treated Aged SAMP8 Mice

**Figure 2:**
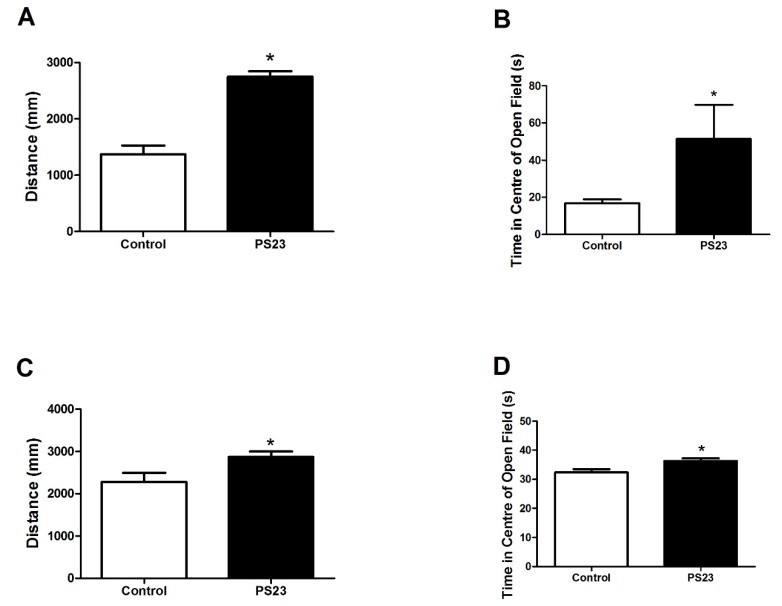
Results of open field test in the control and PS23 groups. (**A**) The total distance moved and (**B**) time spent in the center by the male mice. (**C**) The total distance moved and (**D**) the time spent in the center by the female mice. Data were analyzed using the *t*-test. * *p* < 0.05.

**Figure 3:**
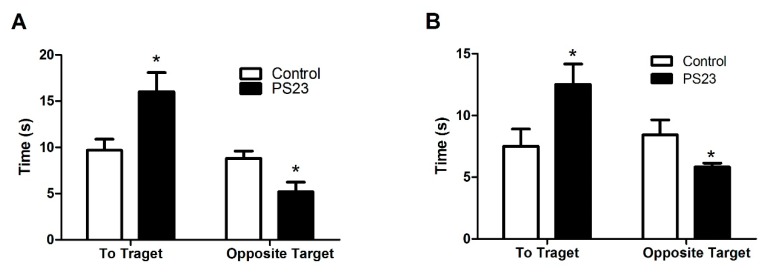
Results of Morris water maze in the control and PS23 groups in the male (**A**) and female (**B**) SAMP8 mice. Data were analyzed using *t*-test. * *p* < 0.05.

The male and female SAMP8 mice treated with LPPS23 were observed to move farther, shown in Figure 2A,C, and stay longer in the center of the OF area, shown in Figure 2B,D, than the untreated mice. The results of the MWM test, shown in Figure 3A, demonstrated that the male LPPS23-treated SAMP8 mice spent 50% more time in the target quadrant and 40% less time in the target-opposite quadrant. The female LPPS23-treated SAMP8 mice spent 90% more time in the target quadrant and 30% less time in the target-opposite quadrant, as shown in Figure 3B.

### 3.3. Neuronal Monoamine Status in the LPPS23-Treated Aged SAMP8 Mice

**Figure 4:**
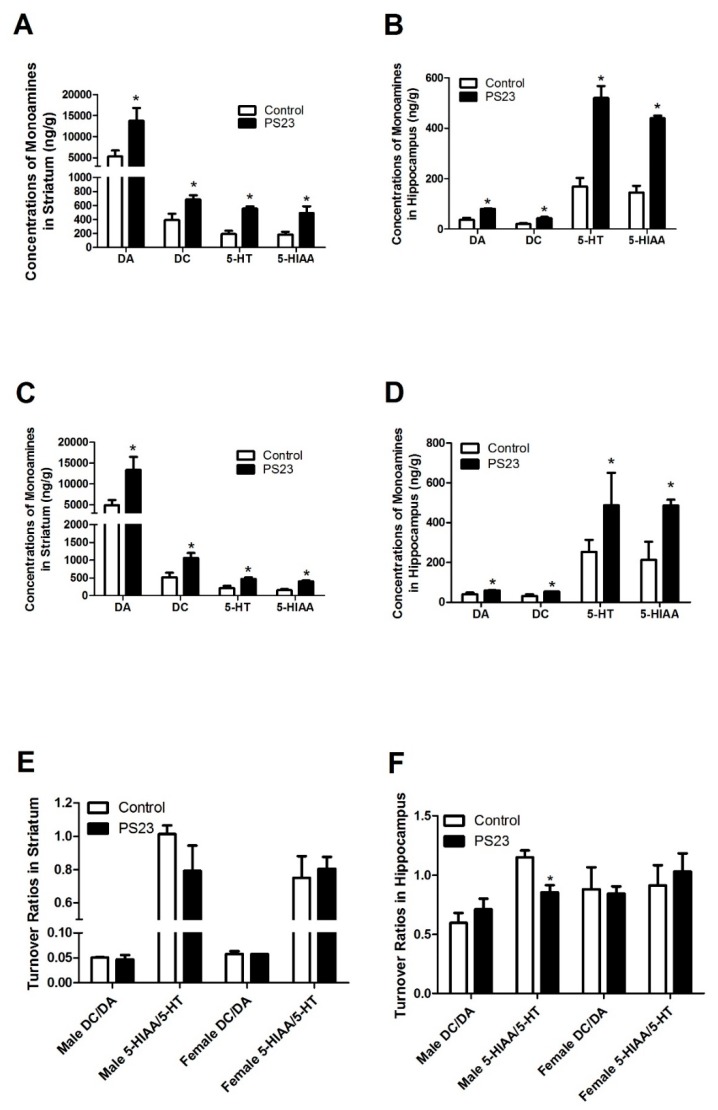
Relative levels of neural monoamines and their metabolites in the striatum and hippocampus of the control and PS23 SAMP8 mice. (**A**) Striatum and (**B**) hippocampus of the male mice. (**C**) Striatum and (**D**) hippocampus of the female mice. Turnover ratios in (**E**) striatum and (**F**) hippocampus. The data were analyzed using the *t*-test. * *p* < 0.05.

**Figure 5:**
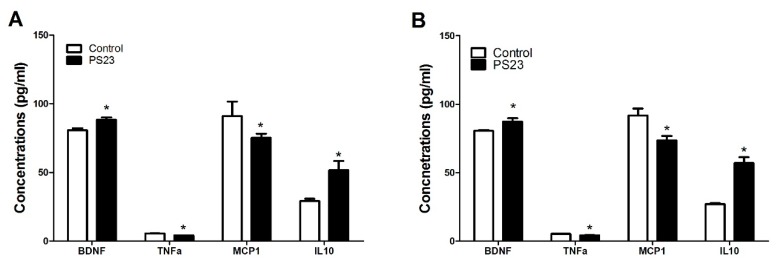
Relative levels of brain derived neurotrophic factor (BDNF), tumor necrosis factor (TNF)-α, monocyte chemotactic protein-1 (MCP-1), and interleukin (IL)-10 in the serum of control and PS23 SAMP8 (**A**) male and (**B**) female mice. The data were analyzed using the *t*-test. * *p* < 0.05.

LPPS23 improved the performance of the mice in the anxiety-like behavioral test (OF test) and memory assessment test (MWM test) therefore, we measured the levels of anxiety- and memory-related neural monoamines in the striatum and hippocampus of the mice. In the striatum, the levels of DA, DC, 5-HT, and 5-HIAA were higher in the PS23 group than in the control group, as shown in Figure 4A,C. Similar trends were observed in neuronal monoamine levels in the hippocampus, as shown in Figure 4B,D. There were no differences in turnover ratios of DC/DA and 5-HIAA/5-HT between the control and PS23 groups in both the striatum and hippocampus, except for the 5-HIAA/5-HT ratio in the hippocampus of male mice, as shown in Figure 4E,F. A low serum level of BDNF is a biomarker of anxiety and memory disorders [35]. The levels of BDNF were 1.1 times higher in the PS23 group than in the control group, as shown in Figure 5. This difference was observed in both the male and female control SAMP8 mice.

### 3.4. Inflammatory and Oxidative Statuses in the LPPS23-Treated Aged SAMP8 Mice

**Figure 6:**
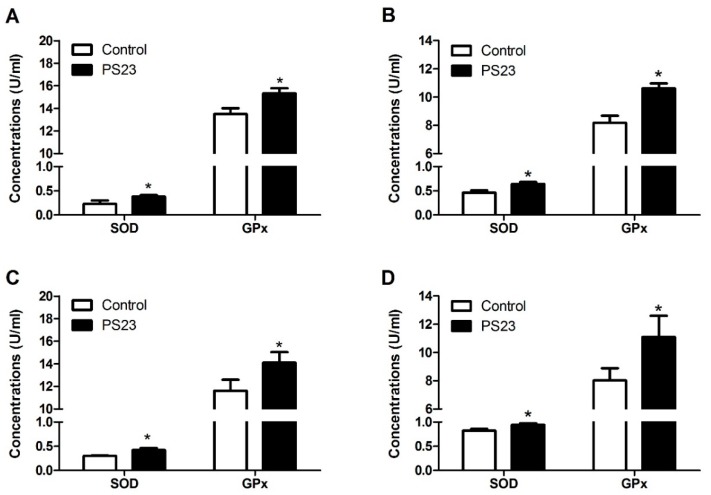
Relative levels of superoxide dismutase (SOD) and glutathione peroxidase (GPx) in the serum of (**A**) male and (**C**) female and in the hippocampus of (**B**) male and (**D**) female mice. The data were analyzed using the *t*-test. * *p* < 0.05.

To determine whether LPPS23 regulated other factors in age-related changes in behavior, the serum levels of TNF-α, MCP-1, and IL-10 were compared between the control and PS23 groups. The lower levels of TNF-α and MCP-1 and the higher levels of IL-10 in the PS23 group than in the control group suggest that LPPS23 attenuated age-related inflammation, as shown in Figure 5. Moreover, the levels of SOD and GPx were measured in the serum, shown in Figure 6A,C, and hippocampus, shown in Figure 6B,D. The higher levels of SOD and GPx in the PS23 group indicated that LPPS23 enhanced the antioxidant capacity in the treated mice.

## 4. Discussion

The present study is the first to reveal the evidence that LPPS23 supplementation at an early stage of aging could delay the early onset symptoms of age-related cognitive dysfunction in SAMP8 mice, such as anxiety-like behaviors and memory impairment. At end of the experiment, the PS23 SAMP8 mice exhibited a lower number of indices of age-related senescence, less anxiety-like behaviors and a lower level of memory impairment than the control SAMP8 mice. These changes were associated with the relatively high levels of neural monoamines in the brain and the high level of BDNF in the serum samples of the PS23 mice. The PS23 SAMP8 mice also showed higher levels of antioxidative enzymes and lower levels of inflammatory cytokines than the control mice. Since LPPS23 improved the age-related criteria in SAMP8 mice, shown in Supplementary Table S1, the results suggest that supplementing LPPS23 during early stages of aging could be a potential strategy for delaying the progression of aging, particularly the onset of anxiety-like behaviors and memory impairment.

Aging is a complex and combinatory process that can result in several systemic and brain disorders [36]. Anxiety is one of the most common age-related issues [37]. Although the prevalence of anxiety is not always linked to aging, several studies have indicated a significant correlation between anxiety and aging [38,39,40,41]. Age-related anxiety is usually caused by dysfunction of the striatum and hippocampus and a decrease in the levels of neural monoamines and BDNF. Thus, modulating age-related alterations in the striatum and hippocampus is necessary for preventing anxiety. Although no studies have previously addressed the effects of probiotics on age-related anxiety, *B. infantis* 35624, *B. longum* NCC3001, and *L. plantarum* PS128 have been reported to reduce a number of anxiety-like behaviors and restore monoamine levels in key brain regions through the gut–brain axis in a maternal separation model of anxiety [12,20,33]. In this study, we evaluated the effects of LPPS23 on anxiety-like behaviors in a murine model of aging, SAMP8, whose behavioral changes occur as early as four months of age [42]. Our results showed that the control SAMP8 mice exhibited anxiety-like behaviors. and their levels of DA, DC, 5-HT, and 5-HIAA in the striatum and hippocampus and serum BDNF levels were lower than the corresponding levels in the pre-aged mice, as shown in Supplementary Table S2. However, the progress of all the aforementioned age-related alterations was retarded if the mice were treated with LPPS23 during 16–28 weeks of age. Therefore, LPPS23 plays a crucial role in maintaining the emotional behaviors and functions of the brain as it ages in mice. Although the results of the behavioral tests and neural monoamine measurements provided evidence to evaluate senescence, it could also be assessed using β-galactosidase, a biomarker of neuronal senescence. However, the brain samples in this study were too small to perform other assays. Thus, β-galactosidase staining could be done in future studies to gain further understanding of the mechanisms by which LPP23 delays neuronal senescence.

The hippocampus and striatum also play critical roles in the formation of new memories [43,44,45]. A reduction in the levels of 5-HT, DA, and BDNF in aged humans and animals has been associated with memory deficiencies [46,47]. Although several probiotics were reported to enhance memory and the levels of neural monoamines [16], no study focused on the effects of probiotics on age-related memory impairment. In the present study, we demonstrated that long-term supplementation of LPPS23 resulted in a longer staying time in the target quadrant and a shorter staying time in the target-opposite quadrant. LPPS23 treatment also reduced age-related alterations in neural monoamine levels in the hippocampus, striatum, and serum. To our knowledge, LPPS23 is the first strain of probiotics that attenuates the effects of aging on memory and memory-related neural monoamines in the mouse brain.

Since the levels of 5-HT and DA could be influenced by the metabolic rates, we then studied the turnover rate by analyzing the ratios of 5-HIAA/5-HT and DC/DA. The turnover ratio was only significantly different for 5/HIAA/5-HT in male mice between the control and PS23 groups. These results suggest that the high levels of 5-HT and DA in the PS23 group were not because of LPPS23 reducing the turnover rates of 5-HT and DA.

The higher levels of 5-HT and DA in the PS23 group raise the question of whether LPPS23 induced too much 5-HT and DA. High levels of 5-HT can cause serotonin syndrome [48]. The behaviors associated with serotonin syndrome include hind limb abduction, head weaving, Straub tail, tremor, low body posture, backward movement, and forepaw treading [49]. High levels of DA are associated with mental disorders like schizophrenia [50] and bipolar disorder [51]. Although the levels of 5-HT and DA were around two times higher in the striatum and hippocampus of the PS23 group than the control group, the differences were <11% between the PS23 and pre-aged mice. Moreover, no abnormal behaviors were observed in the PS23 group. Therefore, the levels of 5-HT and DA in the PS23 group are not likely to be associated with serotonin syndrome or schizophrenia.

To further investigate how LPPS23 ameliorates age-related anxiety-like behaviors and memory deficits, we analyzed the levels of antioxidant factors and inflammation-related cytokines. Reactive oxygen species (ROS) and peripheral inflammation are highly correlated with brain dysfunction with respect to emotion and memory in aged individuals [52]. SOD and GPx can inactivate ROS and protect brain cells against oxidative stress-induced neurotoxicity [53]. SOD has also been suggested to prevent neurobehavioral damage induced by chronic oxidative stress, which can occur during ageing, and to attenuate age-related cognitive impairments and anxiety [53,54]. This study showed that LPPS23 induces SOD and GPx activity in the serum and hippocampus of aging SAMP8 mice and therefore LPPS23 may have an anti-ROS capability, which in turn reduces age-related anxiety-like behaviors and memory impairment.

Anti-inflammatory action is another possible mechanism that LPPS23 uses to ameliorate age-related anxiety-like behaviors and memory impairment. Similar to ROS, inflammation increases and causes alterations in emotion and memory in elderly individuals [52]. The reduction in the levels of inflammatory cytokines, TNF-α and MCP-1, and the induction of the anti-inflammatory cytokine, IL-10, in the PS23 group indicate that LPPS23 maintained brain function by reducing the levels of inflammation. High anxiety has been associated with a poor memory [55] and therefore LPPS23 may also ameliorate memory impairment by modulating anxiety in the aged SAMP8 mice.

The gut–brain axis is a novel concept which states that the gut microbiota and brain can influence each other [56]. An increasing number of studies have indicated that the composition of gut microbiota is associated with age-related anxiety and memory decline and that probiotics affect emotion and memory through the gut–brain axis [57]. Although the effects of LPPS23 on the intestinal microbiota are unknown, several previous studies have suggested that *L. paracasei* plays a crucial role in microbiota–gut–brain axis modulation [27,58,59,60]. Moreover, our results demonstrated that LPPS23 increased the levels of neural monoamines, such as DA and 5-HT, in the brain and BDNF in the serum, which could be affected by gut microbiota through the gut–brain axis [12]. Furthermore, LPPS23 may also reduce the number of anxiety-like behaviors and memory impairment by altering communication along the gut–brain axis in the aged SAMP8 mice. However, a recent review article suggested that the effects of LPPS23 on the gut microbiome–brain axis may be sex dependent [61]. This is contrary to the results of our study where the effects of LPPS23 on anxiety-like behaviors and memory were similar in both sexes. Therefore, additional studies for determining the composition of gut microbiota and metabolites in male and female SAMP8 mice after LPPS23 treatment are necessary.

Probiotics have been demonstrated to prevent age-related disease and delay the activity of pheromones of senescence [62]. LPPS23 not only alleviated anxiety-like behaviors and memory impairment but also improved the appearance of the aged SAMP8 mice. Moreover, LPPS23 induced the levels of anti-oxidant enzymes and reduced the levels of the enhancers of aging progression, inflammatory cytokines, in serum. Therefore, LPPS23 induces a systemic anti-aging effect in SAMP8 mice and may ameliorate other age-related problems.

LPPS23 is one of lactic acid bacteria that are used as probiotics to develop new food formulations with functional characteristics. The dose of LPPS23 that we used in the present study was obtained from previous studies that demonstrated treating mice with probiotics at 1 × 10^9^ CFU/mouse/day improved either anxiety or memory [12,19,32,33]. Moreover, Wang et al.’s review paper indicated that 10^8^–10^10^ CFU/mouse/day of probiotics showed positive effects on cognitive disorders [16]. Since an effective dose is important for functional food, we attempted to predict the dose for humans based on our results. According to the report from the US Food and Drug Administration, the conversion constant between mice and humans is 0.08 [63]. The average weight of mice was 31.1 g in our study. Assuming a human weight of 60 kg, the dose for humans is 1 × 10^9^ CFU/mouse/day × 1/0.0311 ×  0.08 × 60 = 1.5 × 10^11^ CFU/person/day. Although 1.5 × 10^11^ CFU/person/day is acceptable for human functional food, it is much higher than the doses reported to improve behavior-related psychiatric disorders in 15 human studies (10^9^–4.5 × 10^10^ CFU/person/day) ^16^. Therefore, the conversion constant reported by the USFDA seems to be inappropriate for probiotic supplements. Thus, further study is needed to figure out the effective dose for humans.

In conclusion, our study provided the first evidence that the long-term administration of LPPS23 before aging can significantly delay the progression of age-related cognitive symptoms such as anxiety-like behaviors and memory impairment. These results suggest that LPPS23 acts by reducing the levels of ROS and inflammation and by altering communication through the gut–brain axis to modulate age-related cognitive decline. Furthermore, LPPS23 has the potential to become a functional food in the near future.

## Supplementary Materials

The following are available online at http://www.mdpi.com/2072-6643/10/7/894/s1, Table S1: Criteria for grading score of senescence in mice, Table S2: Behaviours and levels of neural monoamines, antioxidative enzymes, and inflammatory cytokines in pre-aged and aged mice.
