# Supplementary material for: Lactobacillus paracasei PS23 Delays Progression of Age-Related Cognitive Decline in Senescence Accelerated Mouse Prone 8 (SAMP8) Mice
Source: Nutrients. 2018 Jul 12;10(7):894. doi: 10.3390/nu10070894 (PMC6073302; doi:10.3390/nu10070894)
Supplement: Supplementary file 1 [file nutrients-10-00894-s001.pdf]

Supplementary table 1 Criteria for grading score of senescence in mice

Item	Define	Grade 0	Grade 1	Grade 2	Grade 3	Grade 4
I) Behavior<br>1. Reactivity	The most intensive exploratory response observed within 30 seconds	Natural behavior	**A.** Abnormal gait with no lessening of agility and behavior patterns<br>**B.** Restlessness	Definite decrease in agility and behavior patterns	Does not move voluntarily but will move if nudged	Immobile
2. Passivity	Escape reaction from pinching of the nuchal skin or from hanging by the forelimb	Natural escape reaction to plnching	Decrease in escape reaction to plnching	Loss of escape reaction to pinching. Preserved righting reaction to manual turn over	Neither escapes reaction to pinching not righting reaction. Escape reaction to hanging by the forelimb	Escape reaction nil
II) Appearance<br>1. Skin and hair<br>(1) Glossiness	Glossinss	Natural gloss	Decrease in gloss	Complete disappearance of gloss	Complete disappearance of gloss and hair appears dirty	Complete disappearance of gloss and hair looks very dirty
(2) Coarseness	Coarseness of hair on the head, nucha and dorsum determined according to the number of palpable, fine clumps of hair	No coarseness	Coarseness of less than an area of the head	Coarseness of less than doubles the area of the head	Coarseness of less than 3 times areas of the head	Complete disappearance of gloss and hair looks very dirty
(3) Loss of hair	Loss or thinning of hair on the head, nucha and dorsum except for changes due to ulcer or periophthalmic lesions	Neither loss nor thinning of hair	**A**. Loss of hair in less than an area of the head<br>**B.** Thinning of hair in less than 1/2 of the area	**A.** Loss of hair in over one area of the head, less than in 1/4 of total area<br>**B.** Thinning of hair in more than 1/2 of total area	Loss of hair in more than 1/4, in less than 1/2 of total area	Loss of hair in over 1/2 of total area
(4) Skin ulcers	Ulcer or healed ulcer on entire skin except for changes associated with periophthalmic lesions	No evidence of ulcer	Healed ulcer or ulcer with scab	Ulcer without healing tendency, in less than one area of the head	Ulcer without healing tendency in more than one area of the head, in less than 1/4 area of all the skin	Ulcer without healing tendency in more than 1/4 area of whole skin
2.Eyes<br>(1)Periophthalmic Lesions	Catarrhal changes in the periophthalmic area or swelling of the palpebra	No changes	Catarrhal changes limited to periophthalmic area or swelling of palpebra	Catarrhal changes extending to nose	Catarrhal changes extending further	
(2)Corneal pacity	Opaque changes of cornea with rough surface by direct ophthalmoscopy	No opacity	Opacity with visible iris	Opacity with visible iris. Positive retinal reflex by transillumination	Opacity of entire cornea	
(3)Ulcer of the cornes	Opaque changes of cornea with rough surface by direct ophthalmoscopy	No ulcer	Linear ulcer corresponding to palpebral fissure	Extension of ulcer over most of the area	Ulcer of entire cornea	
(4) Cataract	Opaque changes of crystalline lens without retinal reflex by transillumination. Impossible to soccer because of coexistence of grade 3 corneal opacity or ulcer	Natural reflection	Diminished reflection	No reflection		
3.Spine<br>(1)Lordokyphosis of the spine	Examined by inspection and palpation	Natural anteroposterior curvature	Increased curvature disappears with digital pressure on the dorsum	Increased curvature disappears with a combination of manual cephalocaudal traction and digital pressure on the dorsum	Permanent curvature	

Supplementary table 2. Behaviours and levels of neural monoamines, antioxidative enzymes, and inflammatory cytokines in pre-aged and aged (the control group) mice

		Female		Male	
		Pre-aged	Aged	Pre-aged	Aged
Behaviours	OF-distance (mm)	3,737.8±397.3	1,761.7±215.4*	3,557.3±305.6	1,374.7±152.2*
	OF-centre time (s)	46.3±3.9	32.7±1.6*	33.2±3.1	16.8±2.0*
	MWM-target quadrant (s)	12.2±1.5	7.5±1.4*	16.3±2.2	9.7±1.8*
	MWM-opposite quadrant (s)	4.0±1.3	9.4±1.5*	5.2±0.6	8.8±1.1*
Striatum	DA (ng/g)	13,145.2±2,290.2	4902.8±322.5*	13,235.2±447.3	5323.2±556.8*
	DC (ng/g)	629.1±45.3	524.2±27.9*	623.8±25.8	393.3±30.0*
	5-HT (ng/g)	510.0±14.1	214.6±47.4*	544.3±9.2	193.9±14.4*
	5-HIAA (ng/g)	408.8±47.7	152.8±29.5*	491.7±31.0	196.7±19.1*
Hippocampus	DA (ng/g)	86.9±10.9	40.1±8.7*	99.0±4.6	36.7±6.7*
	DC (ng/g)	50.8±4.2	30.8±8.8*	46.5±3.5	20.3±2.1*
	5-HT (ng/g)	437.8±76.3	213.2±52.1*	543.0±26.4	145.6±10.5*
	5-HIAA (ng/g)	437.5±45.1	253.3±62.4*	439.3±25.7	168.8±19.4*
Serum	BDNF (pg/mL)	86.0±0.3	80.6±0.6*	83.6±0.5	80.7±0.8*
	SOD (U/mL)	0.50±0.05	0.30±0.01*	0.58±0.07	0.46±0.05*
	GPx (U/mL)	14.1±0.4	11.6±0.6*	14.7±0.3	13.1±0.3*
	IL-10 (pg/mL)	40.8±1.2	27.0±0.3*	44.8±0.9	29.1±1.0*
	TNF-α (pg/mL)	4.7±0.0	5.2±0.0*	4.8±0.1	5.4±0.1*
	MCP-1 (pg/ml)	56.4±2.8	91.8±5.0*	62.4±4.5	91.0±6.1*

OF-distance, total distance moved in open field test; OF-centre time, centre staying time in open field test; MWM-target quadrant, target-quadrant staying time in Morris water maze test. MWM-opposite quadrant, target-opposite quadrant staying time in Morris water maze test. * Significantly different from the pre-control group at $p < 0.05$.
